# Repetitive transcranial magnetic stimulation improves Parkinson’s freezing of gait via normalizing brain connectivity

**DOI:** 10.1038/s41531-020-0118-0

**Published:** 2020-07-17

**Authors:** Tao-Mian Mi, Saurabh Garg, Fang Ba, Ai-Ping Liu, Pei-Peng Liang, Lin-Lin Gao, Qian Jia, Er-He Xu, Kun-Cheng Li, Piu Chan, Martin J. McKeown

**Affiliations:** 1 Department of Neurology, Neurobiology and Geriatrics, Xuanwu Hospital of Capital Medical University, Beijing Institute for Brain Disorders, Beijing, China; 2 National Clinical Research Center for Geriatric Disorders, Beijing, China; 3 Pacific Parkinson’s Research Centre, University of British Columbia, Vancouver, Canada; 4 Division of Neurology, Department of Medicine, University of Alberta, Edmonton, Canada; 5 Department of Electronic Science and Technology, University of Science and Technology of China, Hefei, China; 6 Beijing Key Laboratory of Learning and Cognition, School of Psychology, Capital Normal University, Beijing, China; 7 Department of Radiology, Xuanwu Hospital of Capital Medical University, Beijing, China; 8 Clinical Center for Parkinson’s Disease, Capital Medical University, Beijing, China; 9 Key Laboratory for Neurodegenerative Disease of the Ministry of Education, Beijing Key Laboratory for Parkinson’s Disease, Beijing, China; 10 Department of Medicine (Neurology), University of British Columbia, Vancouver, Canada

**Keywords:** Parkinson's disease, Parkinson's disease

## Abstract

Robust, effective treatments for Parkinson’s freezing of gait remain elusive. Our previous study revealed beneficial effects of high-frequency rTMS over the supplementary motor area. The present study aims to explore the neural mechanisms of rTMS treatments utilizing novel exploratory multivariate approaches. We first conducted a resting-state functional MRI study with a group of 40 Parkinson’s disease patients with freezing of gait, 31 without freezing of gait, and 30 normal controls. A subset of 30 patients with freezing of gait (*verum* group: *N* = 20; *sham* group: *N* = 10) who participated the aforementioned rTMS study underwent another scan after the treatments. Using the baseline scans, the imaging biomarkers for freezing of gait and Parkinson’s disease were developed by contrasting the connectivity profiles of patients with freezing of gait to those without freezing of gait and normal controls, respectively. These two biomarkers were then interrogated to assess the rTMS effects on connectivity patterns. Results showed that the freezing of gait biomarker was negatively correlated with Freezing of Gait Questionnaire score (*r* = −0.6723, *p* < 0.0001); while the Parkinson’s disease biomarker was negatively correlated with MDS-UPDRS motor score (*r* = −0.7281, *p* < 0.0001). After the rTMS treatment, both the freezing of gait biomarker (0.326 ± 0.125 vs. 0.486 ± 0.193, *p* = 0.0071) and Parkinson’s disease biomarker (0.313 ± 0.126 vs. 0.379 ± 0.155, *p* = 0.0378) were significantly improved in the *verum* group; whereas no significant biomarker changes were found in the *sham* group. Our findings indicate that high-frequency rTMS over the supplementary motor area confers the beneficial effect jointly through normalizing abnormal brain functional connectivity patterns specifically associated with freezing of gait, in addition to normalizing overall disrupted connectivity patterns seen in Parkinson’s disease.

## Introduction

Freezing of gait (FOG), characterized by sudden and brief episodes of inability to produce effective forward stepping, is one of the most common and debilitating symptoms in Parkinson’s disease (PD). It is a major risk factor for falls and contributes greatly to reduced mobility and quality of life^1^. Treatment of FOG is very challenging, as evidence for pharmacological treatment, deep brain stimulation, and rehabilitation strategies is inconclusive^2^. Repetitive transcranial magnetic stimulation (rTMS), a noninvasive neural modulation technique, has been used as a treatment for various neurologic and psychiatric disorders^3^. A recent meta-analysis carried out by Wagle et al. ^4^ demonstrated that rTMS therapy improves general motor symptoms and can be used as a potential adjunct therapy for PD patients. However, it is suggested that future studies are warranted to specifically examine rTMS effects on particular clinical features of PD, including FOG.

There is increasing evidence suggesting that supplementary motor area (SMA) plays an important role in the pathogenesis of FOG^5–7^, and may be a potential rTMS treatment target. In our previous study, we therefore investigated the clinical efficiency of high-frequency rTMS over SMA on FOG in PD patients. The detailed protocol can be found in our pervious publication^8^. Briefly, we performed a randomized, double-blind, sham-controlled experiment with a parallel design consisting of two arms: 10-Hz rTMS over SMA and *sham* stimulation. Thirty eligible and willing PD-FOG subjects were randomly assigned (with a 2:1 ratio) into two groups with sealed envelopes, to receive either a *verum* (*N* = 20) or *sham* (*N* = 10) rTMS protocol. V*erum* or *sham* rTMS over SMA were carried out in 10 sessions over two successive weeks, one session per day for 5 consecutive days per week. A 7-cm, handheld, figure-of-8 coil was connected to a biphasic magnetic stimulator (Magstim Rapid; The Magstim Co. Ltd., UK). In each session, a 5-s burst of 10-Hz rTMS was repeated every minute for 20 times (in total, 1000 pulses, 20 min duration). Medication was kept constant throughout the trial, all interventions and assessments were carried out in the “ON” state at approximately the same time of day. The improvement of FOGQ score was used as the primary clinical outcome; with MDS-UPDRS III and a Timed Up-and-Go (TUG) test as secondary clinical outcomes. With a 4 week’s follow-up, our results revealed a significantly decreased FOGQ score, significant improvements of MDS-UPDRS III and gait variables in the verum group; whereas no significant improvements were found in the sham group^8^.

Though our prior study investigated the clinical effects of rTMS therapy on FOG in PD patients, however, the underlying neural mechanisms of rTMS-induced improvements need to be further addressed. Resting-state functional MRI (rs-fMRI) proves valuable in understanding the pathophysiology of some features of PD, including FOG^9–11^. This non-invasive method infers neural activity from spontaneous blood-oxygen-level-dependent (BOLD) signal fluctuations^12^. Functional connectivity can be inferred from spatially distinct brain regions that show temporally correlated time courses during rest^13,14^. In this study, we aim to explore the neural mechanisms of rTMS treatments utilizing rs-fMRI techniques. More specifically, in order to establish if rTMS over SMA works on FOG specifically or on PD generally (or both), we applied novel exploratory multivariate approaches to identify the imaging biomarkers for FOG (FOGbm) and PD (PDbm) by contrasting the functional connections in datasets from PD patients with FOG (PD-FOG) to those without FOG (PD-noFOG) and normal controls (NC), respectively. Next, we studied the effects of high-frequency rTMS on the aforementioned two imaging biomarkers. We hypothesized that at least one biomarker would be improved, which would indicate that the abnormal brain connectivity pattern of PD-FOG was normalized or modulated by the utilization of rTMS over SMA.

## Results

### Participants

**Fig. 1:**
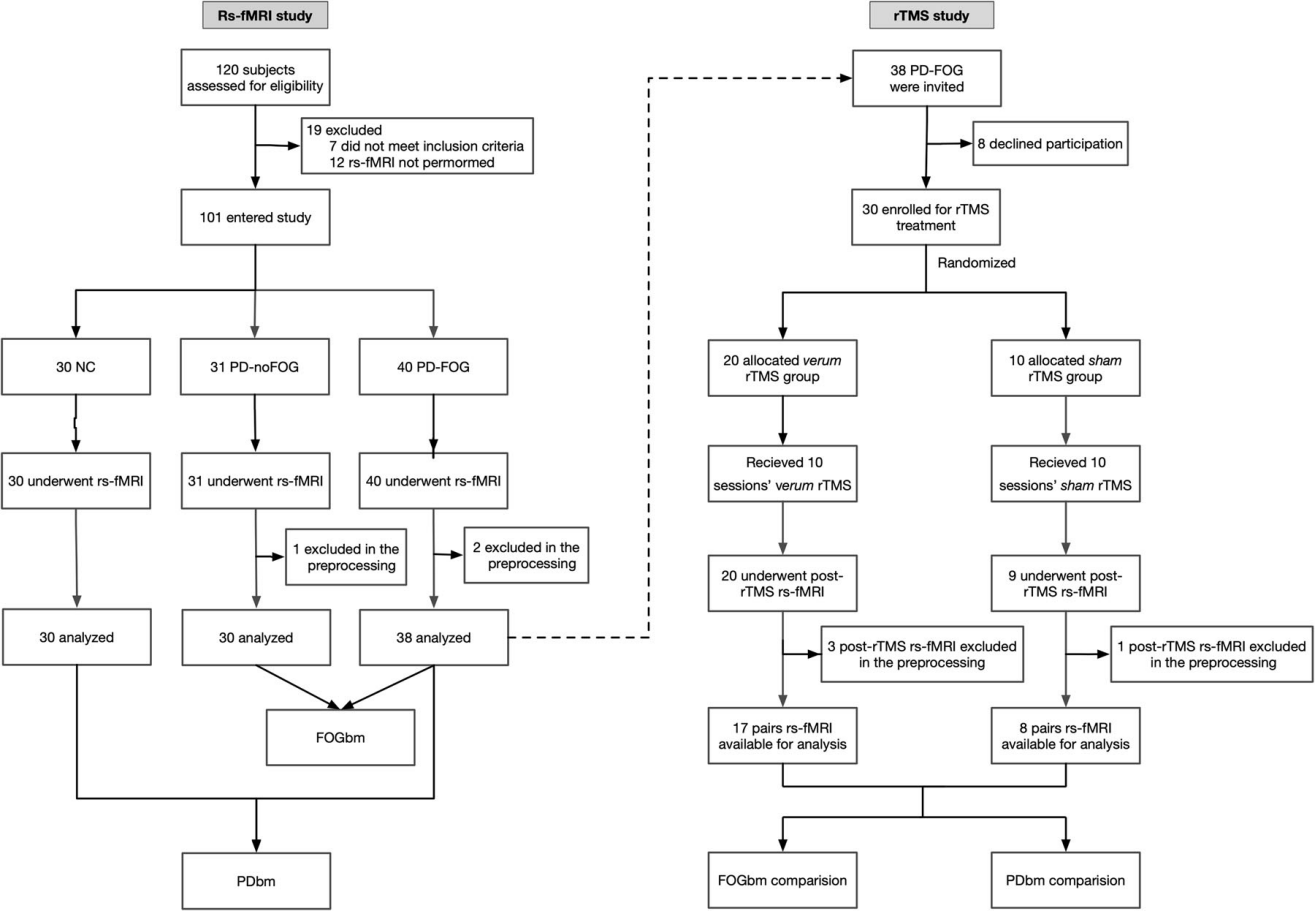
Flowchart of the participants of the rs-fMRI study and the rTMS study.

The flow of participants is presented in Fig. 1. In the rs-fMRI study, two PD-FOG and one PD-noFOG subjects were excluded in the preprocessing due to the failure of the automatic segmentation and overall poor data quality. Thus rs-fMRI data from 38 PD-FOG, 30 PD-noFOG, and 30 NC were ultimately analyzed. In the rTMS study, the post-rTMS fMRI of three patients in the *verum* group and one in the *sham* group were excluded during preprocessing. In addition, there was also one patient from the *sham* group who could not perform the post-rTMS fMRI scan for personal issues. Therefore, a total of 17 pairs of pre-rTMS and post-rTMS fMRI comparisons in the *verum* group and 8 pairs in the *sham* group were finally analyzed.

Participant demographics and clinical features are described in Table 1. Briefly, PD-FOG subjects had longer disease duration, more severe parkinsonism as assessed by H-Y stage and MDS-UPDRS III scores, as well as higher levodopa equivalent daily dose (LEDD). There were no significant differences in gender, age, and MoCA scores among the three groups. In addition, patients in the *verum* and *sham* group had similar baseline characteristics.

**Table 1:** Demographic and clinical features of participants.

Variables	rs-fMRI study			*p*	rTMS study		*p*
	PD-FOG (*N* = 40)	PD-noFOG (*N* = 31)	NC (*N* = 30)		*verum* rTMS (*N* = 20)	*sham* rTMS (*N* = 10)	
Gender (female/male)	20/20	11/20	16/14	0.4530	11/9	5/5	0.7958
Age (years)	62.03 ± 9.17	58.03 ± 9.78	58.30 ± 7.46	0.1075	62.65 ± 10.56	65.60 ± 8.68	0.4241
Disease duration (years)	8.18 ± 5.07	5.23 ± 3.46	–	0.0049*	9.15 ± 5.82	7.40 ± 4.83	0.3932
Onset side (B/R/L)	7/21/12	3/19/9	–	0.6054	3/12/5	2/7/1	0.6203
H-Y stage	2.58 ± 0.78	1.90 ± 0.61	–	0.0001*	2.60 ± 0.85	2.35 ± 0.91	0.4802
MDS-UPDRS III (OFF)	42.23 ± 18.56	30.81 ± 14.41	–	0.0047*	–	–	–
MDS-UPDRS III (ON)	–	–	–	–	34.15 ± 13.60	35.30 ± 16.71	0.8529
LEDD (mg/d)	698.8 ± 398.3	398.3 ± 291.3	–	0.0007*	759.5 ± 458.4	637.2 ± 434.3	0.4838
FOG subtype (OFF/OFF-ON freezer)	29/11	–	–	–	15/5	7/3	0.7703
FOGQ	16.00 ± 4.64	2.45 ± 1.73	–	<0.0001*	15.85 ± 4.87	14.70 ± 4.03	0.4999
MoCA	25.18 ± 3.90	25.13 ± 3.51	26.13 ± 3.52	0.4749	25.10 ± 4.61	25.50 ± 4.35	0.8185

Means and SD are shown for continuous variables.

*FOG* freezing of gait, *Onset side (B/R/L)* bilateral/right/left onset, *H-Y stage* Hoehn and Yahr stage, *MDS-UPDRS III* Movement Disorder Society-Unified Parkinson’s Disease Rating Scale motor score, *FOGQ* freezing of gait questionnaire, *LEDD* levodopa equivalent daily dose, *MoCA* Montreal Cognitive Assessment.

*p < 0.01.

### rTMS study: clinical efficiency

A more detailed description of the clinical efficiency of rTMS can be found in our previous publication^8^. Briefly, the patients were assessed at baseline (*T*_0_), after the 5th (*T*_1_) and 10th (*T*_2_) sessions, and then 2 weeks (*T*_3_) and 4 weeks (*T*_4_) after the last session. As shown in Table 3, with a 4 weeks follow-up, our results revealed significant interaction effects between group (*verum* group and *sham* group) and time (*T*_0_, *T*_1_, *T*_2_, *T*_3_ and *T*_4_) in the FOGQ (*p* = 0.04), MDS-UPDRS III (*p* = 0.02) and several gait variables (total duration, *p* < 0.01; cadence, *p* = 0.04; turn duration, *p* = 0.01; and turn to sit duration, *p* = 0.02). Post-hoc analyses showed that in the *verum* group, FOGQ score was significantly decreased at *T*_2_ and *T*_4_, while MDS-UPDRS III and gait variables were significantly improved at *T*_1_, *T*_2_, *T*_3_, and *T*_4_. However, no significant improvements were found in the *sham* group. Regarding the adverse effects, three subjects in the *verum* group and one subject in the *sham* group reported mild but tolerable headache.

### Rs-fMRI study: FOGbm and PDbm identification

**Fig. 2:**
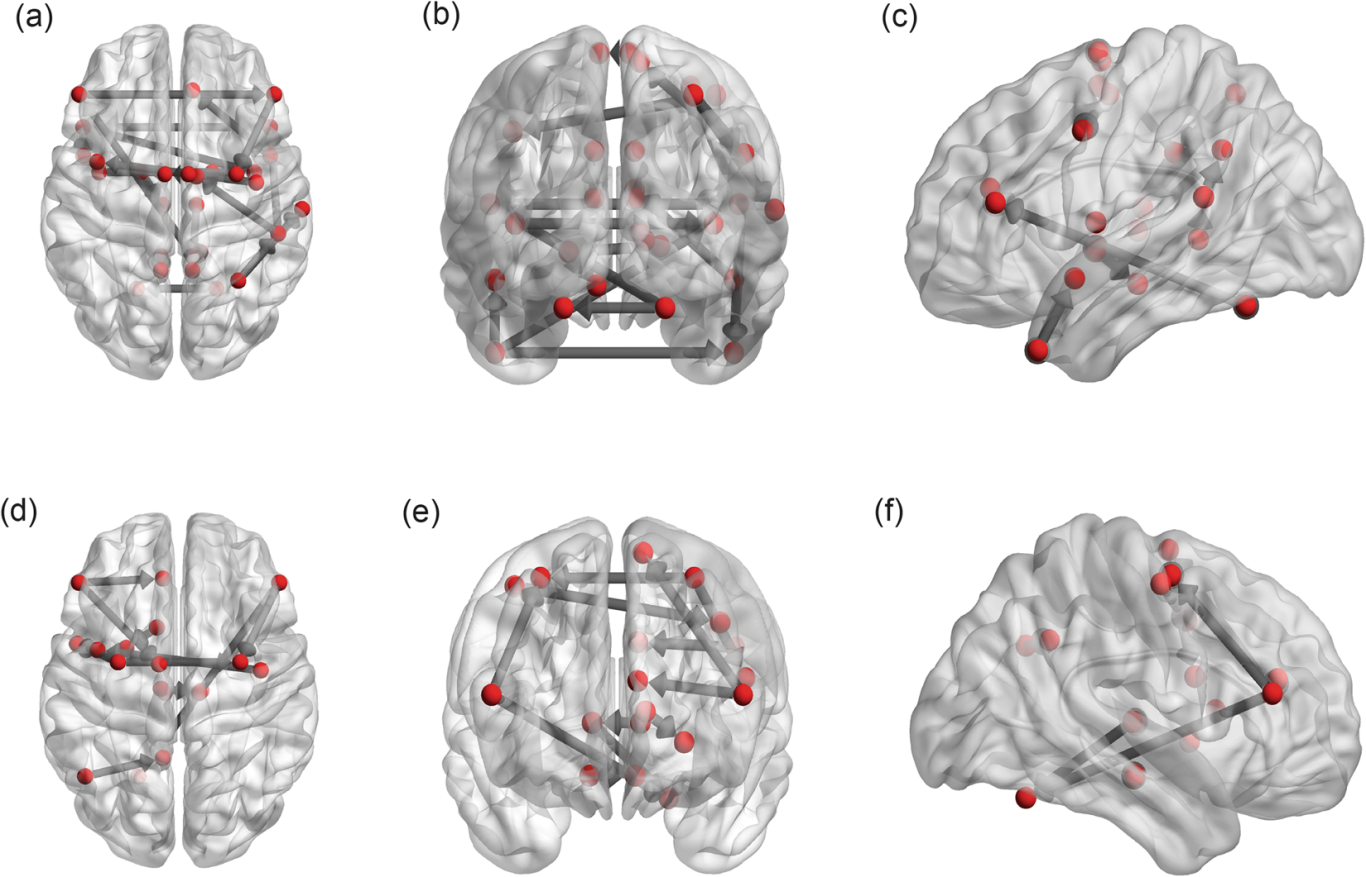
The individual directional connections within the selected network found to significantly predict FOGbm and PDbm. **a**–**c** FOGbm: from the horizontal, coronal, and sagittal plane view; **d**–**f** PDbm: from the horizontal, coronal, and sagittal plane view.

The PCfdr method detected 160 significant connections between the selected ROIs, representing ~6.5% of all possible 50 × 49 = 2450 directional connections. When computing the FOGbm and PDbm, 20 and 12 out of these 160 functional connections, respectively, survived after the LASSO regression operator. Their directional connections and signs are shown in Table 4 (Fig. 2a–f).

**Fig. 3:**
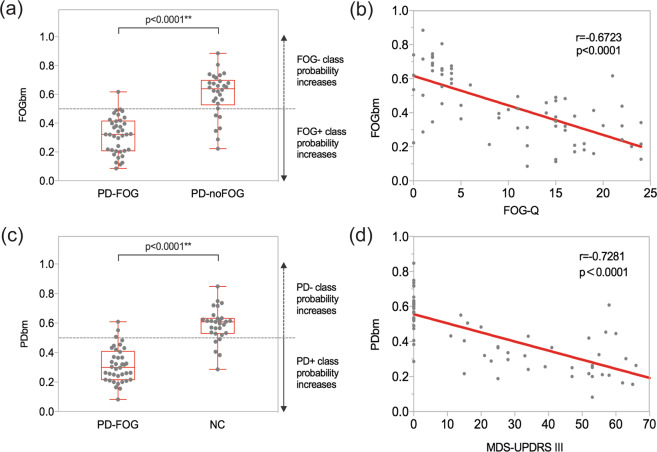
Group difference and correlation with clinical scores of FOGbm and PDbm. **a** FOGbm difference between PD-FOG and PD-noFOG. **b** Correlation between FOGbm and FOGQ scores. **c** PDbm difference between PD-FOG and NC. **d** Correlation between PDbm and MDS-UPDRS III scores. Each dot represents every single patient.

Using the FOGbm, 97.4% (37/38) PD-FOG and 20% (6/30) PD-noFOG were identified as FOG+ (*χ*^2^, *p* < 0.0001), providing a sensitivity of 97.4%, specificity of 80.0%, positive predictive value of 86.0% and negative predictive value of 96.0%. Mean FOGbm of PD-FOG was significantly smaller than PD-noFOG (0.316 ± 0.127 vs. 0.600 ± 0.153, *p* < 0.0001) (Fig. 3a). Correlation analysis between FOGbm and FOGQ scores showed a significantly negative correlation (*r* = −0.6723, *p* < 0.0001) (Fig. 3b).

Ninety-two percent (35/38) PD-FOG and 16.7% (5/30) NC were considered as PD+ (*χ*^2^, *p* < 0.0001) by PDbm, giving a sensitivity of 92.1%, specificity of 83.3%, positive predictive value of 87.5% and negative predictive value of 89.3%. PD-FOG had smaller mean PDbm than NC (0.315 ± 0.117 vs. 0.587 ± 0.114, *p* < 0.0001) (Fig. 3c). Also, there was a negative correlation between PDbm and MDS-UPDRS III scores (*r* = −0.7281, *p* < 0.0001) (Fig. 3d).

### rTMS study: FOGbm and PDbm comparison

At baseline, the *verum* and *sham* groups had similar values of FOGbm (0.326 ± 0.125 vs. 0.333 ± 0.176, *p* = 0.9171), as well as similar PDbm (0.313 ± 0.126 vs. 0.313 ± 0.105, *p* = 0.9986) before stimulation. All 17 patients in the *verum* group and 87.5% (7/8) patients in the *sham* group were identified as FOG+ using FOGbm. Ninety-four percent (16/17) patients in the *verum* group and all of the eight patients in the *sham* group were identified as PD+ using PDbm.

**Fig. 4:**
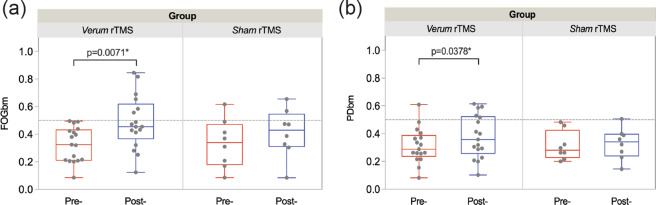
Biomarker changes between pre-rTMS and post-rTMS. **a** Significantly increased FOGbm after the *verum* stimulation (left), whereas no significant change was found after the *sham* stimulation (right). **b** Significantly increased PDbm after the *verum* stimulation (left), whereas no significant change was found after the *sham* stimulation (right). Each dot represents every single patient.

After *verum* stimulation, both FOGbm (0.326 ± 0.125 vs. 0.486 ± 0.193, *p* = 0.0071) and PDbm (0.313 ± 0.126 vs. 0.379 ± 0.155, *p* = 0.0378) were significantly increased (Fig. 4a-left and b-left). However, neither of these two biomarkers showed significant differences after *sham* stimulation (0.333 ± 0.176 vs. 0.409 ± 0.176, *p* = 0.1117; 0.313 ± 0.105 vs. 0.328 ± 0.112, *p* = 0.3518) (Fig. 4a-right and b-right). LASSO regression showed that the greater improvement in FOGbm could be predicted by shorter disease duration and less LEDD, as well as greater improvements in MDS-UPDRS III and turn duration; in contrast, no clinical scores could predict the difference of PDbm.

Qualitatively, six patients considered as FOG+ at baseline were converted into the FOG− group after *verum* stimulation (McNemar’s test, *p* = 0.0412); there were two patients who converted into the FOG− group after the *sham* stimulation, however, this was not significant (McNemar’s test, *p* = 0.4795). The “transferred” and “non-transferred” patients in the *verum* group had similar clinical features except that the transferred subjects had shorter disease duration (6.33 ± 3.39 vs. 11.00 ± 6.51 years, *p* = 0.0355) as well as lower MDS-UPDRS III scores (23.50 ± 7.63 vs. 33.90 ± 12.18 points, *p* = 0.0479). There were four patients in the *verum* group (McNemar’s test, *p* = 0.1336), as well as one in the *sham* group (McNemar’s test, *p* = 1.000) that moved from the PD+ to the PD− group after stimulation, but neither of these were statistically significant.

## Discussion

The present study is the first to explore the underlying brain connectivity mechanisms in supporting the potential treatment effects of rTMS on FOG utilizing rs-fMRI. To this end, we carried out a rs-fMRI study and developed two imaging biomarkers, FOGbm and PDbm, which were negatively correlated with the severity of FOG and motor symptoms, respectively, suggesting that they could be used as biomarkers to assess the effects of rTMS on connectivity patterns. In the subsequent rTMS study, our results revealed significantly increased FOGbm and PDbm after *verum* stimulation, demonstrating that high-frequency rTMS over SMA not only improves the brain connectivity pattern specifically associated with FOG but also the pattern associated with PD overall. These results therefore suggest that high-frequency rTMS over SMA could alleviate FOG via normalization of abnormal brain connectivity patterns.

We found an imaging biomarker (FOGbm) that contributes to our understanding of the physiopathological basis of FOG. Functional imaging techniques have previously demonstrated that during “motor arrests”, PD-FOG has decreased neural activity within the bilateral sensorimotor regions and a concomitant increased response within fronto-parietal cortical regions^7,15^. Another rs-fMRI study demonstrated that functional connectivity disruption of the “fronto-parietal” network is associated with the development of FOG in PD patients^16^. Decreased neural responses have also been observed in a number of subcortical nuclei within the frontostriatal loops during FOG episodes, including the bilateral caudate head, thalamus, subthalamic nucleus, and globus pallidus internus^15^. A re-organization of functional communication within the locomotor network, including the SMA, subthalamic nucleus, mesencephalic, and cerebellar locomotor region, has also been demonstrated in PD-FOG^17^. Taken together, what emerges from these studies is that frontoparietal and frontostriatal dysfunction, as well as neural alterations within the locomotor network, may all take part in the pathophysiology of FOG^1^. In the present study, we selected the union of all these brain regions as our ROIs.

We identified 20 functional connections between brain regions where the connection strengths significantly predicted PD-FOG from PD-noFOG (FOGbm). Consistent with what has been previously described, our results also demonstrated altered functional connectivity within frontoparietal and sensorimotor regions. The increased connectivity from the pallidum to the PPN in PD-FOG subjects that we observed is in accordance with the “interference model” of FOG^18^, which proposes that FOG is triggered by a paroxysmal excessive inhibition of PPN induced by increased inhibitory outputs originating from the pallidum. In addition, we observed that PD-FOG subjects also had increased connectivity strengths within the superior temporal gyrus and insula. The superior temporal gyrus is closely linked with the prefrontal cortex and amygdala^19^, which is involved in emotional processing and notably the triggering of fear responses^20^. The insula is related to autonomic changes^21^. We speculate that the increased connectivities we observed involving these regions are related to fear of falling in PD patients, which is more common and remarkable in those with FOG^22^. We also found that PD-FOG subjects had significantly increased functional connectivity between the precuneus and posterior cingulate cortex, which are involved in the default mode network. We suggest that the increased connectivity between these two regions may reflect a compensatory effect, as these regions exhibited decreased gray matter volume in a previous voxel-based morphometry study^23^. Additionally, previous voxel-based morphometry studies have reported reduced gray matter volumes in the inferior frontal gyrus, precentral gyrus, inferior and superior parietal gyrus, precuneus, thalamus, and PPN among PD-FOG subjects relative to both PD-noFOG subjects and healthy controls^5,23–25^, the increased connectivities among these regions may be reflective of compensatory effects. We notice that there is not notable presence of SMA in the FOGbm. As the number of connectivity features is huge (2450 directional connections), only a sparse set of features were selected which represents the optimal combination of these features. We think this is due to the possible collinearity existing among features, the single significant connectivity feature or important SMA feature may not be selected as it can be represented by a number of other connectivity features and they are better in predicting/associating with the disease.

Several mechanisms could explain the favorable effect of rTMS on PD-FOG. First, the FOGbm, which represents the specific abnormal brain connectivity pattern in PD-FOG from those without FOG, is significantly improved after *verum* stimulation, but not *sham* stimulation. Moreover, this difference was correlated with clinical improvements. These results suggest that rTMS over SMA changes the brain connectivity pattern of PD-FOG towards the pattern of PD-noFOG, in other words, it, at least partly, works on normalizing disrupted connectivity patterns seen in FOG specifically. Previous animal^26,27^ and human^28,29^ studies have revealed that rTMS over cortex can confer effects on remote subcortical regions. Therefore, we assume that the beneficial effect of rTMS over SMA may attribute to its modulation on the brain connectivity evolving other remotely connected brain regions. However, given the fact that rTMS induces neuronal excitability^30^, we cannot totally rule out the possibility that it might also work through the facilitation of the underactivated neurons of SMA. Further analyses regarding the specific brain activity changes of SMA after rTMS is warranted. We found that those patients who transferred from FOG+ to FOG− after *verum* stimulation had shorter disease duration than those did not, and FOGbm difference could be predicted with shorter disease duration. These results suggest that patients may benefit more from rTMS at earlier stages of the disease. Similarly, the PDbm was also significantly increased after stimulation in the *verum* group, but not in the *sham* group. Together with the improvement in MDS-UPDRS III scores, as mentioned above, we propose that rTMS over SMA could also influence overall brain connectivity patterns indicative of PD. In addition, we noticed that there were two patients moved from the FOG+ to FOG− group, as well as one patient moved from the PD+ to PD− group after *sham* stimulation. This could be attributed to the placebo effect in PD, which is mediated through substantial release of endogenous dopamine in the striatum^31^, thereby improving general PD symptomatology. We note that although FOG becomes more dopa-resistant with disease progression, it is generally l-dopa responsive in early stages^2^. Thus the dopa-mediated placebo effect may improve FOG symptomatology in early stage patients. In conclusion, we speculate that rTMS over SMA confers not only a direct therapeutic effect but also a placebo effect, both of which can improve abnormal brain connectivity patterns of PD-FOG.

In this study, we utilized the exploratory multivariate approaches instead of looking at each single feature to identify the specific brain connectivity pattern. As the brain is one of the most complex systems, single connectivity changes may be inadequate to represent the whole brain functioning. Current results further indicate that rTMS has wide impacts over the whole brain connectivity which demonstrates the importance in studying the whole brain connectivity patterns using novel exploratory multivariate approaches.

In conclusion, our results suggest that high-frequency rTMS over SMA confers the beneficial effect by normalizing the abnormal brain functional connectivity pattern of PD-FOG and makes it not only more similar to those without FOG, but also more similar to NC. This study provides powerful evidence of high-frequency rTMS over SMA serving as an add-on therapy for alleviating FOG in PD patients.

## Methods

### Participants

The experiments were performed according to the Declaration of Helsinki and were approved by the Institutional Review Board of Xuanwu Hospital of Capital Medical University. Written informed consent was obtained from all participants prior to the study. Patients diagnosed with idiopathic PD according to the UK Brain Bank Clinical Criteria were recruited from the Movement Disorders Clinic of the Xuanwu Hospital between August 2015 and December 2017. Exclusion criteria were: (i) presence of contraindications for rs-fMRI or rTMS; (ii) history of deep brain stimulation surgery; (iii) marked rest tremor; (iv) comorbidities of neurological disease other than PD; (v) history of receiving any kinds of rTMS; and (vi) left-handedness. FOG subjects were identified by three criteria as described in our previous study^8^. In addition, a control group of sex-matched and age-matched healthy volunteers were recruited from the local community or from patients’ relatives. The study was registered at the Clinical Trial Registration (http://www.clinicaltrials.gov, unique identifier: NCT03219892).

According to the above criteria, we recruited 40 PD-FOG, 31 PD-noFOG, and 30 NC for the rs-fMRI study to identify the imaging biomarkers, and then invited the PD-FOG subjects to participate in the aforementioned rTMS study. Thirty out of the 40 PD-FOG from the primary group agreed to participate in the rTMS study^8^. All of the PD-FOG subjects enrolled in the study were either OFF freezers (freezing occurs predominantly or even exclusively in the OFF-state) or OFF/ON freezers (dopamine-resistant and no difference between ON and OFF-state), the identification of which were based on patients’ usual medication. The demographic and clinical characteristics of the participants are shown in Table 1.

### Rs-fMRI study: clinical assessments

For all the PD patients, clinical assessments were evaluated during their practical “OFF” state (withdrawal of anti-Parkinson medications for at least 12 h), including the Movement Disorder Society-Unified Parkinson’s Disease Rating Scale motor scores (MDS-UPDRS III), Hoehn and Yahr (H–Y) stage, FOG Questionnaire (FOGQ), and Montreal Cognitive Assessment (MoCA).

### Rs-fMRI study: Rs-fMRI data acquisition

Imaging was carried out in a SIEMENS Trio 3 T scanner. Participants were instructed to keep their head still and eyes closed during scanning, but not fall asleep. Earplugs and a head coil with foam pads were used to minimize machine noise and head motion. For PD patients, rs-fMRI scans were acquired following a 12-h period of medication withdrawal. Note that the data of the 30 PD-FOG subjects who were also enrolled in the rTMS study were used as pre-rTMS rs-fMRI. For each participant, we also acquired high-resolution T1 weighted anatomical images, and a radiologist assessed the images to exclude participants with space-occupying lesions, stroke, or other pathology. Structural images were acquired using a sagittal magnetization prepared rapid gradient echo three-dimensional T1-weighted sequence (repetition time [TR] = 1970 ms, echo time [TE] = 3.9 ms, inversion time [TI] = 1100 ms, flip angle [FA] = 15°). BOLD images were obtained using the following SE-EPI sequence: repetition time = 2000 ms, echo time = 30 ms, slice thickness/gap = 4.0/0 mm, axial slices = 33 layers, flip angle = 90°, FOV = 256 mm × 256 mm, matrix size = 64 × 64, and scanning time = 8 min.

### Rs-fMRI study: Rs-fMRI data preprocessing

The acquired rs-fMRI data were preprocessed using the AFNI software package^32^. Several pre-processing steps were performed, including de-spiking, slice timing correction, and 3D isotropic reslicing. Any head motion during the scan was removed by performing a rigid body alignment. The corresponding T1 scans were used to automatically segment the brain into different regions of interest (ROIs) using FreeSurfer. Each of the subject structural scan was then registered using rigid registration to the corresponding subject’s fMRI scan. The ROI masks thus obtained in T1 space from FreeSurfer were then projected onto the fMRI space. All the subsequent analyses were done in the individual native space rather than in a common template space to prevent introducing any unwanted distortions in the fMRI data by registration to a common template. Next, several sources of variance such as head-motion parameters, their temporal derivatives, squares of the temporal derivatives, white-matter signal, and CSF-signal were removed using regression. The white-matter and CSF-signal confounding timeseries were obtained by averaging over the voxels segmented and labeled as white-matter and CSF, respectively. All functional images were resampled into 3.0 × 3.0 × 3.0 mm^3^ voxels. The obtained fMRI signal was then detrended to remove any linear and quadratic trends from the signal. After detrending, the signal was spatially smoothed using 6 × 6 × 6 FWHM and finally, it was bandpass filtered between 0.01 and 0.08 Hz as has been previously suggested for rs-fMRI studies^33–35^.

### Rs-fMRI study: connectivity analysis (PCfdr-DBN)

As shown in Table 2, we selected 50 ROIs based on regions involved in the fronto-parietal network, the frontostriatal loop, and the locomotor network, all of which have been previously studied and proven to be associated with FOG in PD^15–17^. All of the ROIs except the bilateral pedunculopontine nuclei (PPN) were automatically segmented by FreeSurfer. Locating the fMRI signals from the PPN required special consideration. The PPN is an elongated neuronal collection in the lateral pontine and mesencephalic tegmental reticular zones. Its long axis roughly parallel to the long axis of the floor of the fourth ventricle, with the nucleus straddling the pontomesencephalic junction extending ~5 mm from the mid-inferior collicular level to reach the rostral pons^36^. Due to the limitation of fMRI spatial resolution and the small size of the PPN, any mis-position of PPN area may produce the misleading results in connectivity estimation. As the fMRI signals have been spatially smoothed, here in this study, we included a relatively large number of voxels to represent PPN areas. We first started with the midbrain and pontine Freesurfer labels. At the midbrain/pons junction we divided the anterior–posterior direction of the midbrain into three equal parts. The central partition area, containing the area between the medial lemniscus and superior cerebellar peduncle, was assumed to contain the PPN^37^. We averaged these voxels within this partition to get the PPN signal.

**Table 2:** The 50 ROIs used in the connectivity analysis.

No.	Brain regions	No.	Brain regions
1	ctx_lh_G_precentral	26	ctx_rh_G_precentral
2	ctx_lh_G_postcentral	27	ctx_rh_G_postcentral
3	ctx_lh_G_front_middle	28	ctx_rh_G_front_middle
4	ctx_lh_G_pariet_inf-Angular	29	ctx_rh_G_pariet_inf-Angular
5	ctx_lh_G_pariet_inf-Supramar	30	ctx_rh_G_pariet_inf-Supramar
6	ctx_lh_G_parietal_sup	31	ctx_rh_G_parietal_sup
7	Left-caudate	32	Right-caudate
8	Left-putamen	33	Right-putamen
9	Left-pallidum	34	Right-pallidum
10	Left-thalamus-proper	35	Right-thalamus-proper
11	Left-cerebellum-cortex	36	Right-cerebellum-cortex
12	ctx_lh_insula	37	ctx_rh_insula
13	ctx_lh_G_temp_sup-G_T_transv	38	ctx_rh_G_temp_sup-G_T_transv
14	ctx_lh_G_temp_sup-Lateral	39	ctx_rh_G_temp_sup-Lateral
15	ctx_lh_G_temp_sup-Plan_polar	40	ctx_rh_G_temp_sup-Plan_polar
16	ctx_lh_G_temp_sup-Plan_tempo	41	ctx_rh_G_temp_sup-Plan_tempo
17	ctx_lh_G_and_S_cingul-Ant	42	ctx_rh_G_and_S_cingul-Ant
18	ctx_lh_G_cingul-Post-dorsal	43	ctx_rh_G_cingul-Post-dorsal
19	ctx_lh_G_cingul-Post-ventral	44	ctx_rh_G_cingul-Post-ventral
20	ctx_lh_G_precuneus	45	ctx_rh_G_precuneus
21	Left-PMd	46	Right-PMd
22	Left-PMv	47	Right-PMv
23	Left-SMA	48	Right-SMA
24	Left-pre-SMA	49	Right-pre-SMA
25	Left-PPN	50	Right-PPN

**Table 3:** Clinical efficiency of the *verum* and *sham* rTMS.

	*Verum* group	*Sham* group		*F*	*p*
FOGQ					
* T*_0_	16.04 ± 0.82	16.00 ± 1.84	Group	0.34	0.56
* T*_2_	14.44 ± 0.82	15.40 ± 1.83	Time	3.04	0.06
* T*_4_	13.91 ± 0.84	16.40 ± 1.84	Group × time	3.57	0.04*
MDS-UPDRS III					
* T*_0_	34.75 ± 3.08	35.40 ± 4.36	Group	0.62	0.44
* T*_1_	31.75 ± 3.08	35.10 ± 4.36	Time	7.12	<0.01*
* T*_2_	29.55 ± 3.08	34.60 ± 4.36	Group × time	3.15	0.02*
* T*_3_	28.06 ± 3.10	33.41 ± 4.37			
* T*_4_	28.96 ± 3.11	35.16 ± 4.39			
Gait analyses					
Total duration (s)					
* T*_0_	26.15 ± 2.16	27.34 ± 3.06	Group	1.44	0.24
* T*_1_	23.11 ± 2.16	28.45 ± 3.07	Time	1.41	0.23
* T*_2_	23.61 ± 2.17	28.13 ± 3.06	Group × time	4.97	<0.01*
* T*_3_	24.24 ± 2.19	29.46 ± 3.08			
* T*_4_	23.70 ± 2.19	29.50 ± 3.10			
Cadence (steps/min)					
* T*_0_	113.73 ± 2.07	122.89 ± 2.92	Group	0.92	0.34
* T*_1_	115.98 ± 2.07	118.24 ± 3.00	Time	0.72	0.58
* T*_2_	115.63 ± 2.09	118.33 ± 2.92	Group × time	2.68	0.04*
* T*_3_	117.16 ± 2.23	119.07 ± 3.09			
* T*_4_	116.40 ± 2.23	114.89 ± 3.20			
Turn: Duration (s)					
* T*_0_	4.01 ± 0.47	4.10 ± 0.66	Group	1.12	0.30
* T*_1_	3.53 ± 0.47	4.61 ± 0.67	Time	0.36	0.83
* T*_2_	3.39 ± 0.47	4.46 ± 0.67	Group × time	3.30	0.01*
* T*_3_	3.59 ± 0.48	4.36 ± 0.67			
* T*_4_	3.54 ± 0.48	4.69 ± 0.68			
Turn to Sit (s)					
* T*_0_	5.73 ± 0.51	5.64 ± 0.72	Group	0.96	0.34
* T*_1_	4.91 ± 0.51	5.98 ± 0.72	Time	0.71	0.59
* T*_2_	4.83 ± 0.51	5.93 ± 0.72	Group × time	3.05	0.02*
* T*_3_	4.99 ± 0.52	6.07 ± 0.73			
* T*_4_	5.15 ± 0.52	6.12 ± 0.74			

Means and SD are shown for continuous variables.

**Table 4:** The individual directional connections within the selected network found to significantly predict FOGbm and PDbm.

	From	To	Sign^a^
*FOGbm*			
1	ctx_lh_G_temp_sup-Plan_tempo	ctx_lh_G_temp_sup-Lateral	−
2	ctx_lh_G_temp_sup-Plan_tempo	ctx_rh_G_temp_sup-Plan_tempo	−
3	ctx_rh_G_temp_sup-Lateral	ctx_rh_G_and_S_cingul-Ant	−
4	ctx_lh_G_cingul-Post-ventral	ctx_lh_G_cingul-Post-dorsal	−
5	Right-Thalamus-Proper	ctx_rh_G_cingul-Post-dorsal	−
6	ctx-rh-precuneus	ctx_rh_G_cingul-Post-ventral	−
7	ctx-lh-insula	ctx-rh-insula	−
8	ctx_rh_G_precentral	ctx_lh_G_precentral	−
9	ctx_rh_G_parietal_sup	ctx_rh_G_postcentral	−
10	R_SMA_proper	L_SMA_proper	−
11	ctx_rh_G_pariet_inf-Supramar	R_Pre_SMA	−
12	Left-Pallidum	L_PPN	−
13	Right-Pallidum	Left-Pallidum	−
14	Right-Cerebellum-Cortex	Left-Cerebellum-Cortex	+
15	ctx_lh_G_temp_sup-Plan_polar	ctx-rh-insula	+
16	Right-Cerebellum-Cortex	ctx_lh_G_front_middle	+
17	ctx_lh_G_front_middle	ctx_rh_G_front_middle	+
18	ctx_rh_G_parietal_sup	ctx_rh_G_pariet_inf-Supramar	+
19	ctx_rh_G_pariet_inf-Supramar	ctx_rh_G_temp_sup-Plan_tempo	+
20	ctx_rh_G_front_middle	R_PMd	+
*PDbm*			
1	Right-Thalamus-Proper	Left-Cerebellum-Cortex	−
2	ctx_lh_G_front_middle	ctx_lh_G_and_S_cingul-Ant	−
3	ctx_lh_G_pariet_inf-Angular	ctx_lh_G_cingul-Post-dorsal	−
4	L_PMd	R_PMd	−
5	ctx_rh_G_front_middle	Left-Cerebellum-Cortex	+
6	Left-Caudate	Left-Putamen	+
7	Left-Thalamus-Proper	Right-Thalamus-Proper	+
8	ctx_rh_G_precentral	ctx_lh_G_precentral	+
9	ctx_lh_G_front_middle	L_Pre_SMA	+
10	ctx_rh_G_front_middle	R_PMd	+
11	L_PMd	L_PMv	+
12	R_PPN	L_PPN	+

^a^+/− indicates the positive/negative weights in the LASSO regression.

The connectivity network between ROIs was first computed with a PCfdr algorithm using mean time courses of selected ROIs^38^. The PC algorithm is an efficient Bayesian learning approach^39^ that infers the interactions between variables (in this case, mean voxel values within ROIs) by detecting the conditional dependence/independence relationships between them. An extension of the PC algorithm, the PCfdr algorithm, controlling the type I error rate individually for each connection and integrates a false discovery rate control procedure into the network learning, is suitable for brain connectivity assessment^40^. The results of applying the PCfdr algorithm is a binary undirected connectivity network which embeds the conditional independence relationships into the skeleton of a graph. To further estimate the connectivity directions and strengths, a dynamic Bayesian network (DBN) learning method was then adopted^41^. We chose DBN modeling as it has a solid basis in statistics and easily incorporates the prior domain knowledge. The directionality was estimated according to the *maximum likelihood* criterion. In this study, all the subjects were used to estimate the structures and directions. Then the coefficients were estimated individually for each subject. As stated previously by Li et al.^42^, when performing group level analysis using DBN for functional connectivity interpretations, there are three generally used approaches: assuming a “virtual-typical subject” that learns a common network (both directionality and coefficients) for all the subjects from different groups; a “individual-structure” approach that learns the individual network for each subject separately; and a “common-structure” approach that imposes the same structure (and directionality), but allows individual parameters to vary. The common-structure approach balances the commonality and diversity among subjects. Additionally, the selection of the group level analysis models is usually scenario-dependent. As we need to evaluate the changes of PD and FOG-associated metrics, it is necessary to fix the structure and directionality for all the subjects for subsequent analysis. Therefore, in the present study, we fixed the structure of the connectivity networks, but allowed the strength of the connections to vary between different groups.

To identify the FOGbm and PDbm, a logistic least absolute shrinkage and selection operator (LASSO) regression with leave-one-out cross-validation method was adopted. The strengths of the detected connections were used as independent variables, and group labels (labeled FOG+ and FOG− group as 0 and 1 for FOGbm; labeled PD+ and PD− as 0 and 1 for PDbm) were treated as the response variables in the regression model. The logistic LASSO would optimally distinguish groups and select the important connectivity features in classification (the features with non-zero regression coefficients). As a result, we could compute the estimated group label as the biomarker for FOG (FOGbm) and PD (PDbm), respectively. When computing the FOGbm, we included MDS-UPDRS III scores as a covariate to minimize any effects of disease severity. By contrasting PD-FOG to PD-noFOG, with a threshold of 0.5, a value of FOGbm ranging from 0 to 0.5 was considered as belonging to FOG+, while a value ranging from 0.5 and 1.0 was considered as belonging to FOG−. Similarly, by comparing PD-FOG with NC, PDbm was computed to give the probability of the subject belonging to the PD+ (range from 0 to 0.5) or PD− (range from 0.5 to 1.0) group. Correlations between the biomarkers and clinical scores were also analyzed.

### rTMS study: FOGbm and PDbm comparison

A post-rTMS fMRI scan was acquired 1 or 2 days after the last session of rTMS. The acquisition paradigm, preprocessing and processing methods were same as used in the prior rs-fMRI study. The FOGbm and PDbm generated in the rs-fMRI study were compared between pre-rTMS and post-rTMS to assess the effects of rTMS on the FOG-related and PD-related brain connectivity patterns in PD-FOG patients, respectively.

### Statistics analysis

Demographic data were presented as mean ± SD for continuous variables. Independent two-sided *t*-test was performed for the comparison of continuous variables, and the *χ*^2^ test was used to compare categorical variables. Two-sided paired *t*-test and McNemar’s test were used to test the biomarker changes before and after rTMS. The threshold for the level of significance was set at *α* = 0.05. All statistical analyses were performed using JMP Pro 12.0 software (SAS Institute Inc., NC). Graphics were created using Prism 7.0.

## Supplementary information

nr-reporting-summary

## Data Availability

The datasets generated during and/or analyzed during the current study are available from the corresponding authors on reasonable request.
